# Characteristics and risk factors of bacterial meningitis caused by *Streptococcus agalactiae, Streptococcus pneumoniae* or *Escherichia coli* in Guangzhou China from 2015 to 2022

**DOI:** 10.3389/fcimb.2022.1092468

**Published:** 2023-01-09

**Authors:** Danchun Chen, Benyu Tang, Ying Li, Kelu Zheng, Xiaojing Li, Wenxiong Chen, Fei Gao, Yuanyuan Gao, Kaili Shi

**Affiliations:** ^1^ Department of Neurology, Guangzhou Women and Children’s Medical Center, Guangzhou, China; ^2^ Department of Pediatrics, The Third Affiliated Hospital, Sun Yat-sen University, Guangzhou, China; ^3^ Clinical Laboratory, Guangzhou Women and Children’s Medical Center, Guangzhou, China

**Keywords:** bacterial meningitis, Streptococcus agalactiae, streptococcus pneumoniae, escherichia coli, infection, drug resistance, risk factor

## Abstract

**Introduction:**

Bacterial meningitis (BM) is an infectious disease with high morbidity and mortality rates in children. Although vaccination has improved prevention of BM, this severe disease continues to cause considerable harm to children across the globe. Several risk factors have been identified for BM, including immune status, age, and sex. However, additional patient and disease information is required in order to better understand the local characteristics, epidemiology and risk factors of BM.

**Methods:**

Here, we collected information from 252 children with BM in the Guangzhou Women and Children Medical Centre medical record database infected with *Streptococcus agalactiae*, *Streptococcus pneumoniae*, or *Escherichia coli* between May 2015 and May 2022.

**Results:**

The three pathogen infected BM cased showed distinct trends during the period, and distribution of three BM pathogens across age groups varied significantly. We reviewed the antimicrobial resistance patterns for each of the pathogens which may direct drug use in BM. Finally, we found blood WBC was a protective factor, while glucose levels in the CFS was risk factor, for the length of hospitalization.

**Discussion:**

Collectively, this study provides multi-parameter characteristics of BM, and potentially guide the drug use.

## Introduction

Pediatric bacterial meningitis (BM) is a rare but serious inflammatory disease of the tissues surrounding the brain and spinal cord. It has high neurological morbidity and mortality worldwide (Chacon-Cruz et al., 2016; Tauzin et al., 2019; Chen et al., 2021; Peng et al., 2021). Compared to meningitis caused by viruses, bacterial meningitis is significantly more severe with a fatality rate around 10% and 1 in 5 left with debilitating complications (https://www.who.int/news-room/fact-sheets/detail/meningitis). Globally, the primary causes of acute pediatric BM are *Streptococcus pneumoniae* (*S. pneumoniae*), *Neisseria meningitidis* (*N. meningitidis*), and *Haemophilus influenzae* type b (Hib) (Li et al., 2018). The pathophysiology of BM includes pathogen colonization of the host, survival within the bloodstream, and invasion into the cerebrospinal fluid (CSF) within the central nervous system. Once the pathogen reaches the CSF, it induces immune activation cascades which eventually cause swelling and brain damage (Van De Beek et al., 2016; Van De Beek et al., 2021).

The rate of BM has deceased over recent years with the growing rate of vaccination and other preventive measures (Makwana and Riordan, 2007; Brouwer et al., 2010; Mount and Boyle, 2017). However, BM continues to cause considerable mortality and morbidity in children throughout the world (Shinjoh et al., 2017; Sadeq et al., 2017). There are several identified risk factors for BM, including the immune status of the individual (Brouwer et al., 2010; Costerus et al., 2016; Van Veen et al., 2016), genetic factors (Brouwer et al., 2009), age (Van De Beek et al., 2012; Bijlsma et al., 2016; Van De Beek et al., 2016), and sex (Dias et al., 2017; Hsieh et al., 2021). The identification of precise risk factor needs further information and analysis.

Currently, the characteristics of BM in children from neonate to adolescence are unclear. Therefore, we performed a retrospective study by collecting data from 2015 to 2022 in order to analyze the population characteristics, clinical features, and pathogen resistance patterns, and analyzed risk factors in the most common BM pathogens in our hospital.

## Materials and methods

### Study population

The data collection method was approved by the ethics committee of Guangzhou Women and Children Medical Center.

Data were collected from children with BM in the Guangzhou Women and Children Medical Center medical record database between May 2015 and May 2022.

Inclusion criteria: (1) Hospital admission between May 1, 2015 and May 31, 2022. (2) Children aged 1 day to 18 years old with confirmed BM in our center. (3) *Streptococcus agalactiae* (GBS), *Streptococcus pneumoniae* (*S. pneumoniae*), or *Escherichia coli* (*E. coli*) isolated from the CSF.

Exclusion criteria: (1) Diagnosis with non-bacterial infectious meningitis or meningoencephalitis (viral, tuberculous, fungal, leptospiral, or primary amoebic). (2) Definitive diagnosis of autoimmune encephalitis. (3) Definitive diagnosis of neoplasm involving the nervous system. (4) Patient discharged voluntarily. (5) Insufficient data. Patients meeting any of the above criteria were excluded.

### Data management and statistical analysis

A total of 252 BM cases were initially collected, but after ruling out cases that met any exclusion criteria and confirming the remaining cases according to the WHO standard (http://whqlibdoc.who.int/hq/2011/WHO_IVB_11.09_eng.pdf), we analyzed 173 cases. All data were statistically analyzed by SPSS 26.0 (SPSS Inc. Chicago, IL, USA). To explore risk factors for length of hospitalization with encephalitis, the length of hospitalization was used as the dependent variable, and variables such as age, gender, type of culture bacteria were included sequentially to establish a generalized linear model for univariate analysis, where the independent variables were included in the multivariate analysis model according to the criterion of P<0.1. The multivariate analysis model was developed to explore the risk factors for length of hospitalization in pediatric patients with encephalitis, using the length of hospitalization as the dependent variable and the variables screened by the univariate analysis model as the independent variables. Gamma distribution with log link function in the generalized linear model was used to explore risk factors for the length of hospitalization.

## Results

### Demographic characteristics

From May 1, 2015 to May 31, 2022, 252 patients (<18 years old) with BM were enrolled for screening. The most common BM-associated pathogens isolated were *Streptococcus agalactiae* (GBS), *Streptococcus pneumoniae* (*S. pneumoniae*), and *Escherichia coli* (*E. coli*). In accordance with the exclusion criteria, 173 confirmed BM patients were ultimately included. 60.1% of the enrolled patients were male (104/173) and 39.8% were female. More than half of the patients (50.9%) were between the ages 29 days and 1 year, and 82.7% of patients were <1 year old. There was a sharp decline in BM occurrence with increasing age; no children in this study were older than 12 years old. The most commonly isolated pathogen was *S. pneumoniae*. 89.6% of patients were born at term, and 67.6% were born through the vaginal route. 97.1% of the children came from Guangdong province (**Table 1**).

**Table 1:** Comparison of patient characteristics diagnosed with different etiologies of bacterial meningitis.

	*E. Coli* (n=54) n=54	Spn (n=60) n=60	GBS (n=59) n=59	All cases (n=173) n=173
Sex, n (%)				
Female	14 (25.9)	22 (36.6)	26 (44.1)	69 (39.8)
Male	40 (74.1)	38 (63.3)	33 (55.9)	104 (60.1)
Age				
≤28 days	23 (42.6)	0	31 (52.5)	55 (31.8)
29 days-1 years	31 (57.4)	30 (50.0)	28 (47.5)	88 (50.9)
1-3 years old	0	14 (23.3)	0	14 (8.1)
3-7 years old	0	11 (18.3)	0	11 (6.4)
7-12 years old	0	5 (8.3)	0	5 (2.9)
GA at birth, wk				
≥37	47 (87.0)	58 (96.7)	50 (84.7)	155 (89.6)
32–36+6	5 (9.3)	2 (3.3)	6 (10.2)	13 (7.5)
28–31+6	0	0	3 (5.1)	3 (1.7)
<28	2 (3.7)	0	0	2 (1.2)
Mode of delivery				
Vaginal	46 (85.2)	43 (71.7)	35 (59.3)	117 (67.6)
Cesarean delivery	8 (14.8)	17 (28.3)	24 (40.7)	56 (32.4)
Native place				
Guangdong	52 (96.3)	58 (96.7)	58 (98.3)	168 (97.1)
Other	2 (3.7)	2 (3.3)	1 (1.7)	2 (2.9)

E. coli, Escherichia coli; Spn, Streptococcus pneumoniae; GBS, Group B Streptococcus; GA, gestational age.

### Clinical features

**Figure 1:**
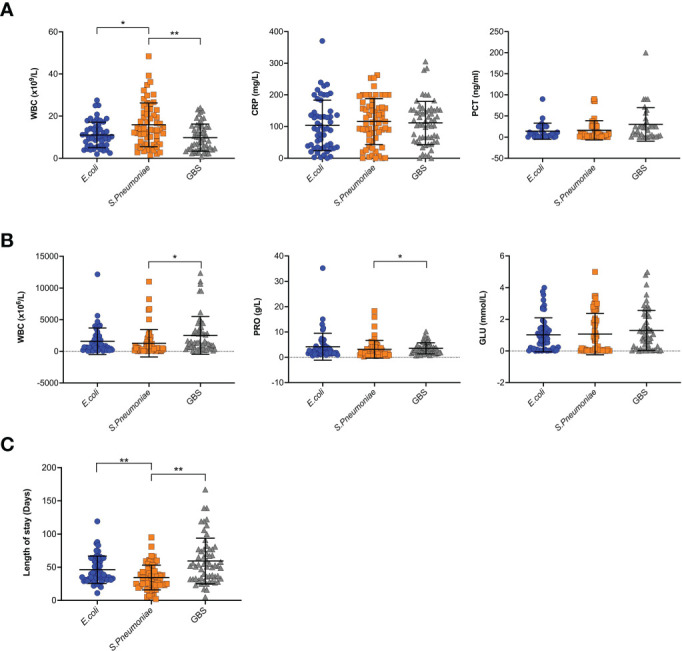
Clinical features of BM. **(A)** Blood samples were tested for WBC, CRP and PCT, and results were shown for each individual. **(B)** CSF samples were tested for WBC, PRO, and GLU, and results were shown for each individual. **(C)** The length of stay in hospital for each patient were shown. (*p<0.05; **p<0.01).

**Figure 2:**
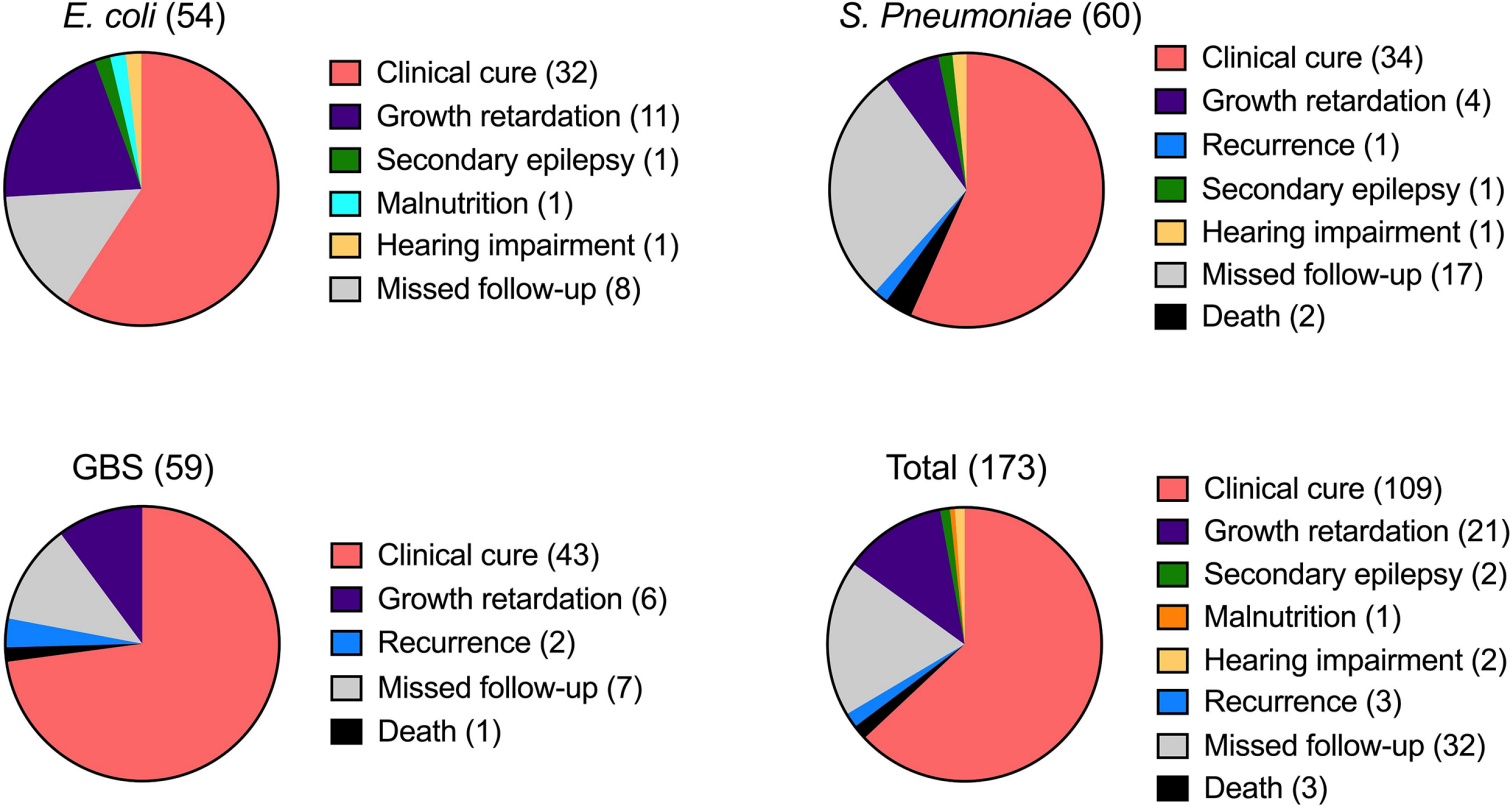
Clinical outcomes of BM. The outcome of BM infection was recorded and percentage of the indicated sign were shown for each infection. The number of cases was shown in brackets.

The average length of inpatient stay was 46.68 days, and the patients with GBS BM had the longest hospitalization lengths (P<0.05). The most common symptom was fever (89.1%), and more than half of the patients had no outward clinical signs. However, patients with *S. pneumoniae* had a higher probability of exhibiting pathological signs (45.0%, P<0.05). Almost all patients had elevated leukocytes (WBC), rapid C-reactive protein (CRP), and procalcitonin (PCT) levels in the blood, as well as high leukocyte and protein levels but low glucose in the CSF. Interestingly, SPn infected patients showed higher WBC levels in the blood (**Figure 1A**), however, in the CSF, WBC and PRO in GBS infected patients showed higher than those in SPn ones (**Figure 1B**). Meningeal enhancement was the most frequent feature of head MRI. Moreover, E. coli and GBS infected patients had longer stay than that of SPn infected ones (**Figure 1C**). The most common complications among all patients were subdural effusion (SE), vision (abnormal visual evoked potential) and hearing impairment; 43.9% of the enrolled patients had no complications following infection (**Table 2**). The percentage of the clinical outcomes were shown in **Figure 2**.

**Table 2:** Comparison of clinical features of patients diagnosed with different etiologies of bacterial meningitis.

	*E. coli* (n=54) n=54	Spn (n=60) n=60	GBS (n=59) n=59	All cases (n=173) n=173	P^1^	P^2^	P^3^	P ^t^
Symptom (n%)					–	–	–	0.102
fever	48 (88.9)	57 (95.0)	52 (88.1)	157 (90.8)				
seizures	13 (24.1)	11 (18.3)	7 (11.9)	31 (17.9)				
other	5 (9.3)	2 (3.3)	5 (8.5)	12 (6.9)				
Sign					0.036	0.003	0.002	0.000
pathological signs (+)	13 (24.1)	27 (45.0)	13 (22.0)	53 (30.6)				
negative	32 (59.3)	29 (48.3)	46 (78.0)	107 (61.8)				
other	9 (16.7)	4 (6.7)	0 (0)	13 (7.5)				
Blood culture (%)	24 (44.4)	30 (50.0)	35 (59.3)	89 (51.4)				0.276
Head MRI					–	–	–	0.236
Meningeal enhancement	42 (77.8)	41 (68.3)	48 (81.4)	131 (75.7)				
SE	18 (33.3)	11 (18.3)	12 (20.3)	41 (23.7)				
Normal imaging	3 (5.6)	9 (15.0)	5 (8.5)	17 (9.8)				
Other	6 (11.1)	9 (15.0)	4 (6.8)	19 (11.0)				
Complication					–	–	–	0.158
SE	17 (31.5)	9 (15.0)	10 (16.9)	36 (20.8)				
V/H impairment	7 (13.0)	13 (21.7)	14 (23.7)	34 (19.7)				
Hydrocephalus	7 (13.0)	12 (20.0)	4 (6.8)	23 (13.3)				
Other	4 (7.4)	3 (5.0)	5 (8.5)	12 (6.9)				
None	19 (35.2)	29 (48.3)	28 (47.5)	76 (43.9)				

E. coli, Escherichia coli; Spn, Streptococcus pneumoniae; GBS, Group B Streptococcus; MRI, magnetic resonance imaging; SE, Subdural effusion; V/H impairment, Visual or hearing impairment. P^1^, E. coli vs Spn; P^2^, E. coli vs GBS; P^3^, E. Spn vs GBS; P^t^, p value of three groups.

### Distribution of major BM pathogens according to particular year and age

**Figure 3:**
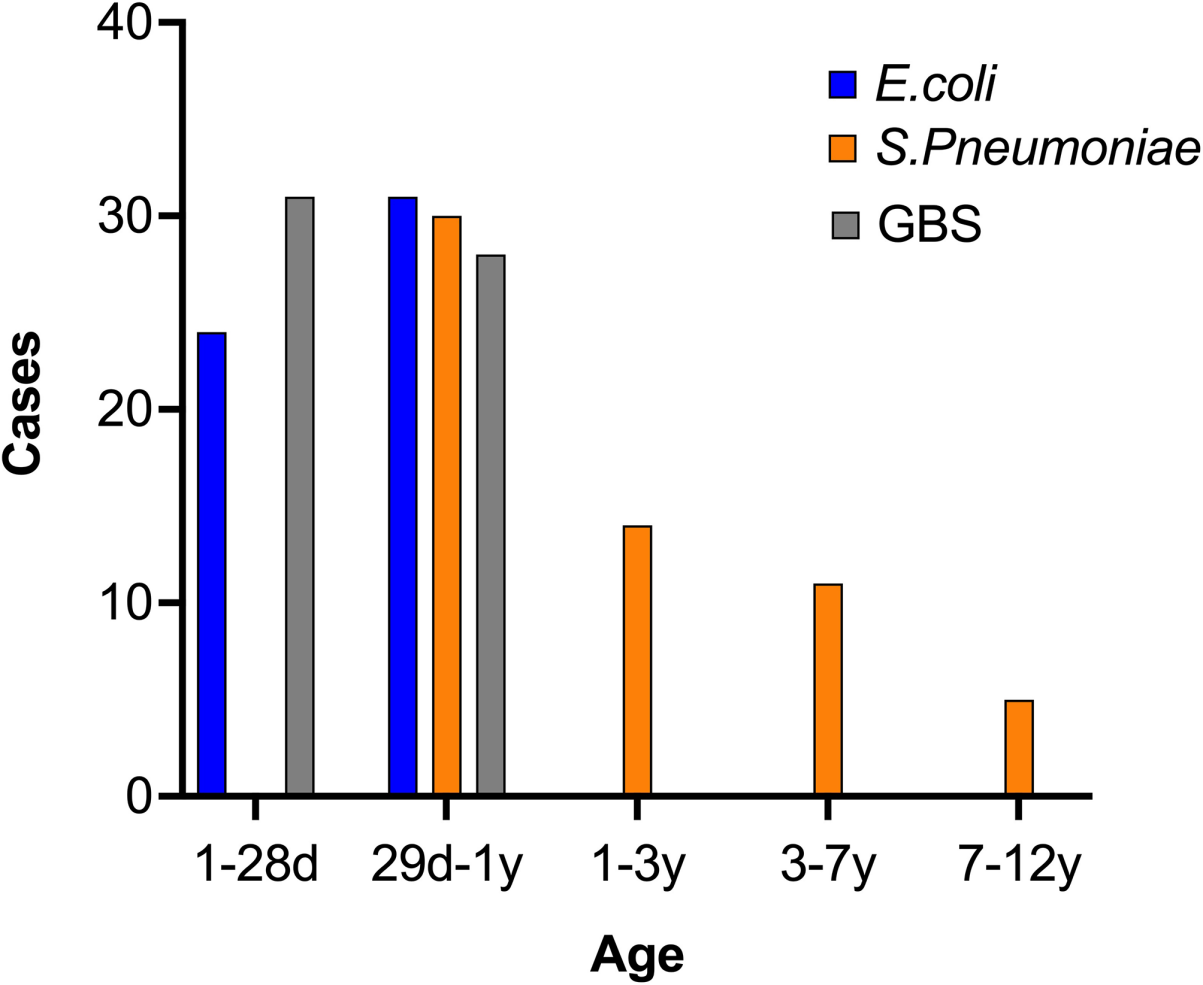
Distribution of three BM pathogens by age. The information of BM patients was collected and the age was analyzed. The case number in each age range was calculated and shown.

**Figure 4:**
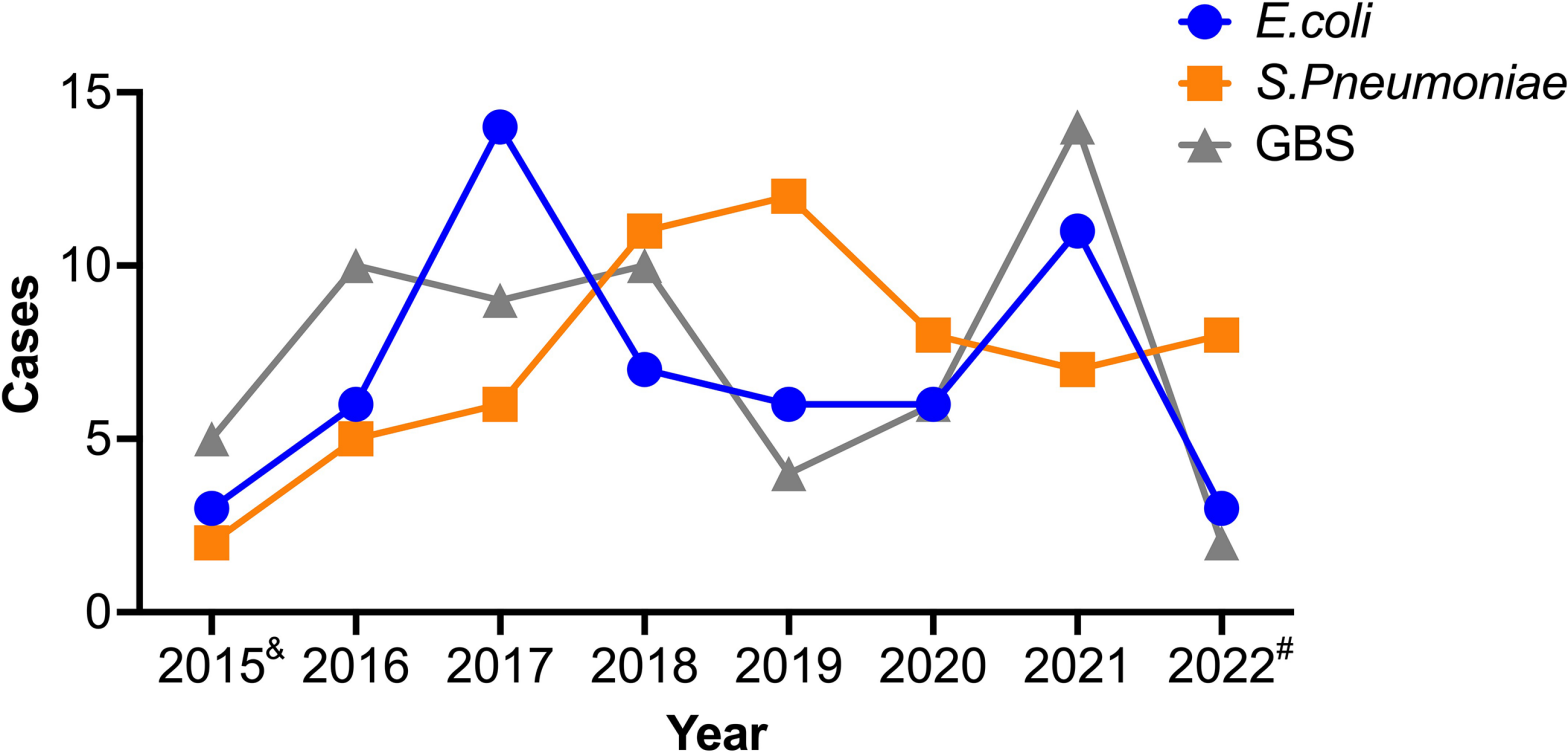
Trends of three etiological causes of BM. The information of patients was collected and the case number in each year was analyzed for the three infectious BM. &, data represent May to Dec of 2015. #, data represent Jan to May of 2022.

To investigate the potential correlation between age and the etiological agent of BM, we calculated the different infecting pathogens across the various age groups. Interestingly, GBS and *E. coli* were commonly reported in patients younger than 1 year, but were rare in older patients, indicating a possible preference of these pathogens for infants (**Figure 3**). Conversely, *S. pneumoniae* was not reported in patients aged 0-1, however it was often found in those over 1 year, indicating that GBS and *E. coli* have divergent infection features relative to *S. pneumoniae*. To account for the possibility that outbreaks of each bacteria and/or the susceptibility of humans change over time, we further studied the diagnosis frequency of each pathogen from 2015-2022. Interestingly, the three pathogen infected BM cases showed distinct trends during the period. E. coli and GBS infected cases showed similar trends, with peaks in 2017 and 2021, while Spn infected cases reached the peak at 2019 (**Figure 4**).

### Resistance rates (%) of the three primary etiological agents of BM

We next evaluated the drug-resistance assays performed on the isolated bacteria to determine the therapeutic approaches for the three bacterial agents. Fresh CSF samples were cultured on a series of antibiotic-coated plates and bacterial growth was monitored. GBS was resistant to Levofloxacin, Tetracycline, and Clindamycin (Resistance rates >10%); *S. pneumoniae* was resistant to Sulfamethoxazole, Cefatriaxone, Azithromycin, Clarithromycin, Erythromycin, Tetracycline, Meropenem, Penicillin, and Cefotaxime; *E. coli* was resistant to Ampicillin, Sulfamethoxazole, Levofloxacin, Cefatriaxone, Ciprofloxacin, and Gentamicin (**Table 3**). Collectively, these drug resistance assays potentially directed the antimicrobial therapy used to treat the patients.

**Table 3:** Resistance rates (A/B) (%) of the three common bacteria isolated from BM patients.

Antimicrobial agent	*E. coli* (n=54)	Spn (n=60)	GBS (n=59)
Amoxicillin	1 (1.9)	5 (8.3)	
Ampicillin	20 (37.0)		
Sulfamethoxazole	28 (51.9)	15 (25.0)	1 (1.7)
Levofloxacin	18 (33.3)		10 (16.9)
Cefatriaxone	6 (11.1)	15 (25.0)	
Cefazolin	3 (5.6)		
Ciprofloxacin	7 (13.0)		
Aztreonam	2 (3.7)		
Gentamicin	6 (11.1)		2 (3.4)
Cefuroxime	5 (9.3)		
Cefepime	2 (3.7)		
Ceftazidime	2 (3.7)		
Azithromycin	–	39 (65.0)	1 (1.7)
Clarithromycin	–	40 (66.7)	
Erythromycin	–	47 (78.3)	
Tetracycline	–	42 (70.0)	28 (47.5)
Meropenem	–	12 (20.0)	
Penicillin	–	43 (71.7)	
Cefotaxime	–	14 (23.3)	
Clindamycin			25 (42.4)
Moxifloxacin			3 (5.1)
Ciprofloxacin			4 (6.8)

A/B (%), number resistant/number tested (percentage resistant); BM, bacterial meningitis; E. coli, Escherichia coli; Spn, Streptococcus pneumoniae; GBS, Group B Streptococcus.

### Risk factors for length of hospitalization

As shown in **Table 4**, using univariate analysis, we demonstrate the risk factors for length of hospital stay in children with encephalitis. Following the criteria of *P*<0.1, we included variables such as gender, type of culture organism, age, perinatal abnormalities, WBC of routine examination, GLU of cerebrospinal fluid examination, and cephalometric imaging in the multivariate analysis model.

**Table 4:** Inivariate analysis of risk factors for length of hospitalization in children with encephalitis.

Variables	*β (SE)*	*P*	*OR (95%*CI*)*
Gender	-0.17 (0.09)	0.056	0.84 (0.71-1.01)
Type of culture bacteria (N,%)			
Escherichia coli	Ref		
Streptococcus pneumoniae	0.29 (0.10)	0.004	1.29 (1.06-1.57)
Group B Streptococcus	0.26 (0.10)	0.011	0.75 (0.61-0.91)
Age (N,%)			
<28 day	Ref		
29 day-1year	-0.16 (0.09)	0.101	0.61 (0.48-0.78)
>1year	-0.50 (0.13)	<0.001	0.86 (0.71-4.03)
Mode of delivery (N,%)	0.14 (0.09)	0.134	1.15 (0.96-1.38)
Full-term birth (N,%)	0.07 (0.14)	0.640	1.07 (0.81-1.42)
Perinatal abnormalities	0.21 (0.10)	0.045	1.24 (1.01-1.52)
Main symptoms (N,%)			
No fever	Ref		
Fever	0.229 (0.16)	0.165	1.10 (0.81-1.50)
Fever + convulsions and other symptoms	0.10 (0.16)	0.539	1.24 (0.92-1.69)
Neurological examination	-0.14 (0.11)	0.205	0.87 (0.71-1.08)
Routine examination			
WBC	-0.34 (0.10)	0.001	0.71 (0.59-0.87)
CRP	0.05 (0.17)	0.784	1.05 (0.76-1.45)
PCT			
Normal	Ref		
Abnormal	0.04 (0.18)	0.806	1.08 (0.76-1.54)
Not done	0.07 (0.18)	0.683	1.04 (0.74-1.48)
Cerebrospinal fluid examination			
WBC	-0.01 (0.19)	0.950	0.99 (0.68-1.43)
PRO	0.29 (0.34)	0.396	1.33 (0.69-2.57)
GLu	0.25 (0.10)	0.016	1.28 (1.05-1.57)
GLU/BG	0.12 (0.15)	0.407	1.13 (0.84-1.51)
Head MRI (N,%)			
No meningeal enhancement	Ref		
Meningeal enhancement	0.28 (0.10)	0.008	1.30 (1.03-1.64)
Meningeal enhancement + effusion and other symptoms	0.26 (0.12)	0.026	1.32 (1.08-1.62)
Blood culture	-0.01 (0.09)	0.922	0.99 (0.84-1.18)

SE, standard error; OR, dominance ratio; CI, confidence interval; WBC, white blood cell; CRP, C-reactive protein; PCT, calcitoninogen; PRO, protein; GLU, glucose; BG, peripheral blood glucose; MRI, magnetic resonance imaging.

As shown in **Table 5**, using multivariate analysis, we demonstrate the risk factors for length of hospitalization in children with encephalitis. We found that absence of GBS (OR=1.27, 95% CI=1.04-1.56) was a risk factor for increased length of hospitalization in children with encephalitis compared to children with culture type Escherichia coli. Abnormal cerebrospinal fluid examination GLU was a risk factor for increased length of hospitalization in children with encephalitis (OR=1.23, 95% CI=1.01-1.49). Compared to children with encephalitis without meningeal enhancement on cranial imaging reality, symptoms such as meningeal enhancement, versus meningeal enhancement and effusion and other symptoms were risk factors for increased length of hospitalization in children with encephalitis (OR=1.23, 95%CI=1.02-1.49; OR=1.35, 95% CI=1.09-1.68,respectively). In addition, abnormal routine examination of WBC was a protective factor for reduced length of hospitalization in the children (OR=0.80, 95% CI=0.66-0.98).

**Table 5:** Multivariate analysis of risk factors for length of hospitalization in children with encephalitis.

Variables	*β (SE)*	*P*	*OR (95%*CI*)*
Gender	-0.10 (0.09)	0.269	0.91 (0.76-1.08)
Type of culture bacteria (N,%)			
Escherichia coli	Ref		
Streptococcus pneumoniae	-0.21 (0.13)	0.090	0.81 (0.63-1.03)
Group B Streptococcus	0.24 (0.10)	0.019	1.27 (1.04-1.56)
Age (N,%)			
<28 day	Ref		
29 day-1year	0.03 (0.11)	0.814	1.02 (0.72-1.43)
>1year	0.02 (0.17)	0.925	1.03 (0.83-1.26)
Perinatal abnormalities	0.07 (0.11)	0.535	1.07 (0.86-1.32)
Routine examination			
WBC	-0.22 (0.10)	0.030	0.80 (0.66-0.98)
Cerebrospinal fluid examination			
GLU	0.20 (0.10)	0.037	1.23 (1.01-1.49)
Head MRI (N,%)			
No meningeal enhancement	Ref		
Meningeal enhancement	0.21 (0.09)	0.028	1.23 (1.02-1.49)
Meningeal enhancement + effusion and other symptoms	0.30 (0.11)	0.005	1.35 (1.09-1.68)

SE, standard error; OR, dominance ratio; CI, confidence interval,WBC, white blood cell; GLU, glucose; MRI, magnetic resonance imaging.

## Discussion

Pediatric bacterial meningitis causes substantial neurological morbidity and mortality across the globe. However, there is limited knowledge regarding the etiology and pathophysiology mechanisms of BM. Although genetic factors associated with the risk of BM have been previously identified, the detailed biological and mechanistic causes of this disease remain elusive. Since patient and disease characteristics vary spatiotemporally, local analyses are required to better identify the features and trends of this substantial disease.

One of the most important findings of this study was that the distribution of the pathogens differ by age, suggesting disparate infection or immunity-based preferences among the observed BM pathogens. Thus, further mechanistic studies are required to better understand the host and pathogen interactions in individuals of different ages. The immune system is founded during early life, thus this critical stage of the growth, immune function development, and especially specific immune cell development and distribution are areas of significant interest with respect to BM.

In our study, Spn infected patients showed elevated WBC levels in the peripheral blood and CSF, however the level of PRO was lower in CSF (**Figure 1A, B**). This provides predictive value for the drug use, before getting the CSF culture results in the clinic. As analyzed for length of hospitalization, E. coli and GBS infected children showed longer stay than those infected with SPn, (**Figure 1C**), suggesting a potential method for prediction for the hospitalization, which is helpful information for the communications with the parents. The analysis of length of hospitalization will also provide hints for the prognosis.

For the risk factor analysis, we found that blood WBC is a protective factor for the length of stay (**Tables 4**, **5**). Given the recognition of WBC levels as an inflammatory sign, we postulate that the immune cells were playing a role in the immune-mediated clearance of pathogens. In the CSF, GLU levels were found to be risk factors for the length of stay (**Table 5**), suggesting a predictive value. GBS infected patients had more hospitalization time (**Tables 4**, **5** and **Figure 1C**), suggesting that GBS infected patients have unique pattern of pathology and need more time for recovery.

A limitation of this study is the lack of genomic data from the patients due to limited genomic and immunological resources. Immune deficient individuals often die from severe infections like BM, thus detailed innate and adaptive immune profiling (e.g., FACS analysis of peripheral blood cells) would be important to combine with clinical data from BM patients to gain better insight into the infection characteristics.

Notably, bacteria change with the transfer of hosts over generations, hence it is reasonable to assume that BM-causing bacteria evolve over time. Therefore, it is important to study the evolutionary aspect of host-pathogen interactions across different BM hosts. This dynamic view of the nature of bacteria would provide significant fundamental knowledge for prevention of specific, BM-causing pathogens.

Taken together, we provide an important clinical resource related to BM patients infected with *Streptococcus agalactiae*, *Streptococcus pneumoniae*, or *Escherichia coli*, especially with respect to incidence trends. This study showed distribution and antibiotic features of isolated pathogens and provided potential hints for drug use in patients with BM.

## Data availability statement

The raw data supporting the conclusions of this article will be made available by the authors, without undue reservation.

## Ethics statement

Data collection method was approved by the ethics committee of Guangzhou Women and Children Medical Center (reference No. 2022-295A01). Written informed consent to participate in this study was provided by the participants’ legal guardian/next of kin.

## Author contributions

DC Collected data, analyzed data and wrote the manuscript. BT, YL, KZ, XL, WC, FG analyzed data. KS wrote the manuscript and organized the study. All authors contributed to the article and approved the submitted version.
